# Parachlamydia acanthamoebae: disease-causing pathogen or opportunistic bystander?

**DOI:** 10.1099/jmm.0.001953

**Published:** 2025-03-19

**Authors:** Simone E. Adams, Carole Kebbi-Beghdadi, Mirja Puolakkainen, Gilbert Greub, on behalf of the ESCMID Study Group for Mycoplasma and Chlamydia Infections (ESGMAC)

**Affiliations:** 1 Institute of Microbiology, Lausanne University Hospital and University of Lausanne, Lausanne, Switzerland; 2 Department of Virology, Helsinki University Hospital, Helsinki, Finland

**Keywords:** *Chlamydia*-related bacteria, *Chlamydiales* order, emerging pathogen, lung infection, protists, virulence

## Abstract

**Introduction.** *Parachlamydia acanthamoebae* is an obligate intracellular bacterium related to disease-causing bacteria like *Chlamydia trachomatis* and *Chlamydia pneumoniae* and is thus classified within the *Chlamydiales* order. *Parachlamydia* was initially discovered within an *Acanthamoeba* strain isolated from water in a humidifier during an investigation of an outbreak of respiratory infections in humans.

**Gap Statement.** The disease-causing potential of this bacterium is not fully understood, but *Parachlamydia* has been associated with bronchiolitis, bronchitis, aspiration pneumonia and community-acquired pneumonia in humans. Additionally, diagnostic testing for *Parachlamydia* infection is not routinely performed, indicating that prevalence is underreported.

**Aim.** This JMM profile aims to gauge what is currently known about the pathogenic potential of *P. acanthamoebae* and bring awareness to gaps in knowledge.

**Results.** Amoebae appear to be the main reservoir of *P. acanthamoebae* and likely enter the nasal passages through contaminated water sources or contact with contaminated animals. The infected amoebae may then descend to the lower respiratory tract where the lytic cycle is triggered, causing human infection.

**Conclusion.** By implementing serology and molecular testing, as well as conducting additional epidemiological studies, a better understanding of the association of human colonization with disease outcomes can be achieved.

## Graphical abstract

*Parachlamydia acanthamoebae* is an obligate intracellular bacterium found in free-living *Acanthamoeba* amoebae. As with other *Chlamydiales, P. acanthamoebae* has a biphasic lifecycle with an infectious elementary body and a replicative reticulate body. Once inside a host, the elementary body differentiates to the reticulate body to undergo replication within the membrane-bound compartment called the inclusion. These infected amoebae can be found in many environments such as in hospitals, household water sources, lakes and farm animals. Although the exact route of transmission from amoebae to humans is unknown, it is believed that humans may breathe in the *Parachlamydia*-infected amoebae. The amoebae can then reside on the respiratory epithelium within the new human host. It is possible that newly released *P. acanthamoebae* can then directly infect human host cells, such as macrophages, pneumocytes or lung fibroblasts, or propagate within infected amoebae. *P. acanthamoebae* has been found in humans with multiple respiratory infections such as pneumonia and bronchitis, indicating an association of the presence of this bacterium with severe disease. Transmission may also occur zoonotically through contact with contaminated animals. The understanding of potential complications of pregnancy associated with *P. acanthamoebae* infection is not understood, but there may be an association with infertility or recurrent miscarriage. Identification and diagnosis can be performed by co-culture of respiratory samples with amoebae or by quantitative PCR using primers specific to *P. acanthamoebae*. This bacterium is found around the world, but due to the lack of testing in patients, the incidence of infection is largely unknown.

## Historical perspective

The first report about *Parachlamydia acanthamoebae* was published in The Lancet in 1997, and its role as a possible pathogen was suspected as the bacterium (Hall’s coccus strain) was discovered living within an amoeba of the *Acanthamoeba* genus [1]. This amoeba was isolated from the water of a humidifier during the investigation of an outbreak of fever in a printshop in the UK. Of note, symptomatic patients who were exposed in the printshop exhibited antibody reactivity against *Parachlamydia*, suggesting a pathogenic role for this strict intracellular bacterium [1]. In the same year, two strains of *Acanthamoeba* were isolated from the nasal mucosa of two human volunteers in Germany [2]. There, the intracellular bacterium *Parachlamydia acanthamoebae* was described. Based on 16S rRNA sequence analysis, both coccoid bacterial parasites (BN9 and Berg17 strains) were related to the *Chlamydia* genus, but too distantly to be a new species in this genus. Therefore, the authors proposed the newly identified bacterium as Candidatus *Parachlamydia acanthamoebae* [2]. The genus name ‘*acanthamoebae*’ refers to the main ecological niche, the *Acanthamoeba*, while the species name ‘*Parachlamydia*’ highlights the morphological similarities with *Chlamydia*, meaning literally ‘beside *Chlamydia*’.

In 1999, Everett *et al*. proposed to classify *Parachlamydia* in the novel family *Parachlamydiaceae* based on phylogenetic analysis of the 16S and 23S rRNA genes [3]. Later, the Pillonel classification, which is based on nine discriminatory core genes, confirmed the assignment of the *Parachlamydia* genus within the family *Parachlamydiaceae* in the *Chlamydiales* order [4].

## Clinical presentation/s

*Parachlamydia* has repeatedly been documented in upper and lower respiratory tract samples including from the sputa, bronchoalveolar lavage and nasopharyngeal samples from humans suffering from bronchiolitis, bronchitis, aspiration pneumonia and community-acquired pneumonia [5]. Multiple intracellular bacteria researchers have noted the pathogenic potential of *P. acanthamoebae* in respiratory infections, but continued research is needed to understand the mode of transmission of * P. acanthamoebae* and its full impact in human and veterinary medicine [6,8]. Some reports also suggest a possible role of * P. acanthamoebae* as an abortive agent in bovines and humans, such as in Finland where *Parachlamydiaceae* DNA was detected in up to 6% of samples from patients with recurrent miscarriage [9,12].

## Microbial characteristics

### Phenotypic features

*P. acanthamoebae*, like the other bacteria in the *Chlamydiales* order, has a biphasic, intracellular lifecycle. These coccoid bacteria have two developmental forms, the smaller (0.2–0.5 µm), infectious elementary body (EB) and the larger (0.5–1 µm), replicative reticulate body (RB) [8,13]. To initiate the infection cycle within its amoebal host, EBs attach to and enter the host cell where they will reside within a membrane-bound compartment termed the inclusion. They will remain within the inclusion for the entire duration of infection. Of note, EBs of *P. acanthamoebae* may exhibit a crescent shape during electron microscopy due to sensitivity to specific fixatives and buffers used during sample preparation [14,15]. Once inside the cell, the EBs differentiate into RBs and begin to proliferate. The newly divided RBs will asynchronously re-differentiate into EBs prior to being released from the host cell. The exit of the bacteria may happen by amoebal lysis or within expelled vesicles [14]. The cycle can then begin again once newly released EBs come into contact with a new cell.

### Genotypic features

With a genome size of ~3.1 Mb (UV7 strain), it is nearly three times larger than its disease-causing relative, *Chlamydia trachomatis* [16,18]. This larger genome size likely aids the bacteria to thrive within free-living amoebae by avoiding the mechanisms that phagocytic protists use to digest engulfed bacteria [19,20]. This large genome may also help the bacteria to endure the variable levels of nutrients available when living in a water-associated, environmental protist [16]. The genome likely emerged through the many opportunities of gene exchange in amoebae, either with other intra-amoebal bacteria and/or with the protist itself [17,21, 22].

One important, horizontally exchanged gene is *katA* which encodes for a functional catalase [23]. This gene allows the bacteria to resist the oxidative stress encountered in the endocytic pathway of amoebae and therefore likely explains the partial resistance to alveolar macrophages. Of note, in human macrophages, the bacteria are trafficked from early endosomes to late endosomes, then preventing the acquisition of lysosomal hydrolases to prevent vacuole acidification by reducing the expression of vacuolar ATPases [24]. This is opposite to *Chlamydia*, which is relocated to the Golgi-associated exocytic pathway [25]. Overall, *P. acanthamoebae* has a more complete metabolic network than other *Chlamydia* species, such as genes necessary to produce menaquinone, a quinone used in the oxidative phosphorylation pathway of most bacteria [16].

Interestingly, *P. acanthamoebae* also possess genes, which encode a chemotaxis system similar to that in *Escherichia coli*. The role of this system during infection is not clear, as there are no flagellar motor proteins to enable cell motility [16]. Finally, similarly to other members of the *Chlamydiales* order, *P. acanthamoebae* encodes the structural components for a type III secretion system (T3SS), which is normally used in all stages of the lifecycle to inject effector proteins into the host [26]. These effector proteins include 38 putative inclusion membrane proteins (Incs), which are embedded into the inclusion membrane and aid in nutrient acquisition and membrane stability, and limit immune detection [27,29]. Although the exact functions of putative Incs in * P. acanthamoebae* have not been determined, it is likely that they have similar functions as Incs in other *Chlamydiales*.

## Laboratory confirmation and safety

### Specimen type

In humans, this bacterium has mainly been documented in upper and lower respiratory tract samples, including nasopharyngeal swabs, sputum, bronchial aspirates and bronchoalveolar lavage [5]. In the future, documentation of the bacterium from a lung biopsy of a patient with an infection will aid in confirming the role of *P. acanthamoebae* as an agent of respiratory disease. As the spectrum of diseases due to *P. acanthamoebae* infection remains unclear, acquiring additional clinical samples will be useful. Samples from urine, placenta, vaginal swabs and semen could be investigated for the presence of the bacterium to understand a possible role in prostatitis, infertility, preterm labour or miscarriage.

### Laboratory confirmation

Diagnosis of *P. acanthamoebae* infection mainly relies on PCR, serology and immunohistochemistry. Recovery of *P. acanthamoebae* by cell culture is only possible by co-culturing samples with amoeba, a technique not performed routinely in diagnostic laboratories [30]. The co-culture can be performed directly from clinical samples using Page’s modified Neff’s amoeba saline as broth. To isolate *P. acanthamoebae*, it is also possible to use amoebal enrichment on non-nutrient agar supplemented with a lawn of Gram-negative bacteria (usually *Klebsiella aerogenes* or *E. coli*), which are provided as a food source for the amoebae. The latter approach was used to isolate the most currently available strains.

To detect the bacteria, a TaqMan quantitative PCR (qPCR) assay was developed by Casson *et al*., which proved to be highly specific to *P. acanthamoebae* and sensitive, with a limit of detection of 10 ml per PCR reaction [31]. Another version of a qPCR assay also exhibited good sensitivity and specificity to *Parachlamydia* [32]. Both of these PCR assays detect the 16S rRNA-encoding gene. Alternatively, the presence of *Parachlamydia* may be suggested in amoebae by Gimenez staining (bacteria are purple on a green background), Diff Quick staining (Dade, Behring, Paris, France) and immunofluorescence with antibodies directed against *Parachlamydia* [33]. However, staining is not specific enough to identify bacteria at the order level, and immunofluorescence may display non-specific interactions.

Diagnosis may also be performed by serology assay. Anti-*Parachlamydia* antibodies can be detected by micro-immunofluorescence by using whole bacteria as an antigen [34]. The cut-off for positivity with IgG is 1 out of 64, whereas the cut-off with IgM is 1 out of 32 [35]. The presence of IgM or a fourfold increase in IgG antibody litres is compatible with a recent infection, whereas IgG may be present from past infections, similar to what is observed for *C. pneumoniae*. Immunohistochemistry may also be used; however, there is significant cross-reactivity with closely related species [36].

Development of an ELISA using recombinant surface proteins was attempted, but with limited accuracy, mainly due to cross-reactivity with antibodies raised by infection with other members of the *Chlamydiales* order [37]. Thus, new approaches such as an ELISA using a mixture of surface proteins obtained by ‘undressing’ the bacterium with detergents are currently in development, as was done earlier for *Waddlia chondrophila* [38].

### Laboratory safety

As *P. acanthamoebae* is a potential emerging pathogen, laboratory handling of the bacterium is performed under biosafety level two (BSL2) conditions. The World Health Organization requires the presence of risk control measures to limit biological risks from the consequences and likelihood of exposure to a pathogen [39]. BSL2 facilities are therefore equipped with biological safety cabinets and air filtration systems to limit exposure to both people within the building and outside of it. These guidelines are suggested in all countries with labs that handle the culturing or isolation of *P. acanthamoebae* species.

## Treatment and resistance

### Treatment

Maurin *et al*. tested the MIC for two strains of *P. acanthamoebae* during infection in *Acanthamoeba polyphaga* [40]. Both strains proved to be susceptible to tetracyclines, macrolides and rifampin but resistant to fluoroquinolones, vancomycin and thiamphenicol. Interestingly, it is also susceptible to gentamicin, while *C. trachomatis* is highly resistant. In 2015, the susceptibility of * P. acanthamoebae* was confirmed, and macrolides were recommended as a treatment for *Parachlamydia*-associated pneumonia, due to the low MIC of 0.06–0.5 µg ml^−1^ [41]. Doxycycline may represent an alternative (2×100 mg day^−1^) in patients with contraindications for macrolides.

### Resistance

Only a limited number of strains have been tested for antimicrobial resistance, but all exhibited resistance to quinolones when tested *in vitro* cell culture. Moreover, all sequenced strains exhibited mutations in the so-called ‘quinolone resistance-determining regions’ (QRDRs) of the DNA gyrase, which encodes genes *gyrA* and *gyrB*, as well as in the DNA topoisomerase IV-encoding genes *parC* and *parE*. Thus, *Parachlamydia* should be considered naturally resistant to quinolones, as the mutations in the QRDR are known in bacterial species (including *E. coli*) to mediate quinolone resistance [42].

## Pathogenic strategies

### Host range

*P. acanthamoebae* was first isolated from *Acanthamoeba* and has been detected both from amoebae residing within humans and from water and soil [1,43]. *In vitro* studies have examined the host range of *P. acanthamoebae* to determine its ability to infect other cells, like human cells and protists other than *Acanthamoeba*, with varying results [6,13, 44]. Human embryonic lung fibroblasts (HEL), human lung carcinoma (A549) and primary macrophages were all permissible to infection by purified * P. acanthamoebae*, when examined for the presence of bacteria by qPCR or confocal microscopy [6,13]. However, the multiplication of *P. acanthamoebae* in macrophages was limited by induced apoptosis [13,45]. Conversely, cervical epithelial HeLa derivative (HEP-2), African green monkey kidney epithelial (Vero), human T lymphocyte (Jurkat), peripheral blood monocyte (THP-1) and phorbol 12-myristate 12-acetate-stimulated THP-1 cells did not show any evidence of infection [44].

Additionally, *P. acanthamoebae* appears to specifically replicate within *Acanthamoebae*, as no bacterial growth was observed following infection of the free-living ciliate, *Tetrahymena thermophila*, or in the myxamoebae, *Dictyostelium discoideum* [44]. Together, these results indicate that the primary host of *P. acanthamoebae* is the protist *Acanthamoeba* but also that there may be potential for this bacterium to infect other cell types under the right conditions. Of note, even if the bacteria are not able to multiply stably within macrophages, the mechanism of induced apoptosis still allows the bacteria to escape from these phagocytic cells and their microbicidal machinery [13,45]. *Acanthamoeba* spp. are implicated in multiple human diseases, and understanding the incidence of *P. acanthamoebae* infection in the environment is important to determine the potential pathogenic role of this bacterium.

### Life cycle

As *Acanthamoeba* spp. are considered to be the reservoir of *P. acanthamoebae*, they may also play a role (i) as a replicative niche and (ii) as a protective armour during the cystic phase [35]. Thus, it is critical to examine their infectious potential towards humans. *Acanthamoeba* spp. are free-living protists that are predominant in the soil and water environments, such as swimming pools or lakes [46]. These protists enter humans either through the eyes, nasal passages or broken skin and have two developmental forms, the cyst and the trophozoite, where the trophozoite is the infectious, replicative form and the cyst is quiescent [47]. These amoebae are able to cause multiple destructive diseases in humans including *Acanthamoeba* keratitis, an eye infection and *Acanthamoeba* pneumonia [48,49]. It is therefore pertinent that testing of *P. acanthamoebae* occurs in coordination with testing for these other amoeba infections. It is crucial that future studies include the detection of both amoebae and the amoebae-resisting chlamydiae to ideally disentangle the respective impact of amoebae and *Parachlamydia* when a symptomatic lung infection is associated with the presence of *Parachlamydia*.

### Virulence factors

Like other members of the *Chlamydiales* order, *P. acanthamoebae* resides within the inclusion, inside of the infected cell. The infectious EB and replicative RB each have effector proteins that interact with the host to maintain its replicative niche. Although little is known about the mechanisms employed by *P. acanthamoebae*, examination of the genome and comparison to other known pathogens like *C. trachomatis* indicate the presence of virulence factors including a *Chlamydia* protease-like activity factor (CPAF) homologue [16]. CPAF is a key *Chlamydiaceae* protease and virulence factor that is secreted into the host cytosol during infection [50]. Interestingly, the absence of the deubiquitinase and deneddylase (ChlaDub2) and immune system evasion protein class I accessible protein-1 (Cap1) indicates the presence of alternative virulence factors in *P. acanthamoebae* that are not present in the *Chlamydia* genus [16].

Another virulence factor that was discovered in the genome of *P. acanthamoebae*, which is encoded by the *dpsA* gene, is a DNA-binding protein implicated in the response to oxidative stress [21]. This gene is also present in *Legionella* species and in *Rickettsia bellii*, a *Rickettsia* previously demonstrated to grow in amoebae. This gene was likely acquired by horizontal transfer through the co-infection of *Acanthamoeba* with other intracellular bacteria such as *Pseudomonas aeruginosa* [51,53]. Survival to the oxidative stress encountered in the endocytic pathway of amoebae, macrophages and neutrophils is facilitated by a bacterial catalase, KatA, that degrades H_2_O_2_ to H_2_O and O_2_. This gene was likely acquired by lateral transfer from gammaproteobacteria [23]. Finally, corruption of the cell host by T3SS effectors likely helps the *Parachlamydia* to inhibit the acidification of the bacterial vacuole and to reduce the level of lysosomal hydrolase within the vacuole [24].

## Epidemiology

### Transmission

Since amoebae are the reservoir for *P. acanthamoebae*, transmission likely occurs from the environment, as human-to-human infection by *Acanthamoeba* has not been thoroughly reported [54]. However, one report examined the potential transmission of *P. acanthamoebae* from a mother to her premature newborn during testing of amniotic fluid by PCR, but there is no widespread evidence of infection occurring via vertical transmission [9]. Fukumoto *et al*. studied smear samples from the floors and sinks in a hospital in Japan for the presence of *P. acanthamoebae* and *Acanthamoeba* DNA, and for the co-occurrence of each [55]. They found a significant correlation between the DNA presence in the smears, indicating that *P. acanthamoebae* likely survives within its amoebal host and that transmission may occur via *Acanthamoeba* by using the protist as a Trojan horse.

### Infection

Once the infected amoeba enters the body, it is likely that it remains on mucosal surfaces, as amoebae infected with * P. acanthamoebae* have been detected from nasal and respiratory tract swabs [16]. *Parachlamydia* seems to follow a lytic exit route in amoebae when the temperature of cell culture is increased from 32 to 37 °C as compared to 25–30 °C [56]. Thus, amoebae present in the nasopharyngeal mucosa may descend to the lungs during bronchoaspiration of secretions (i.e. when a subject is unconscious following trauma). There, the body temperature is typically higher (35 °C) than in the nasal mucosa (25–28 °C), leading to amoebal lysis and the simultaneous release of thousands of bacteria. This lysis will overload the first line of defence, alveolar macrophages, in the lungs and lead to aspiration pneumonia [56].

*In vitro* studies showed that *P. acanthamoebae* is able to infect human macrophages, pneumocytes and lung fibroblasts indicating a further potential to cause respiratory diseases in humans [6,13]. Moreover, animal models have demonstrated that * P. acanthamoebae* may cause pneumonia in mice infected by intratracheal or intranasal inoculation, thus fulfilling the third Koch postulate [57,58].

### Risk groups

Currently, it is not known whether there are certain groups of people that are more at risk of disease than others, but as * P. acanthamoebae* was detected in patients with pneumonia, it is likely that those who are more at risk for pneumonia infection are also more at risk for other opportunistic infections. This includes people with dampened immune systems, the very young and the old [59]. Additionally, people with lung cancer, dementia or ischaemic heart disease are at a greater risk of dying from pneumonia and are therefore more likely to be at risk for *P. acanthamoebae* infection.

### Epidemiology

The epidemiology of *P. acanthamoebae* is not fully understood, as the prevalence of infection in humans with this bacterium is poorly known. Sero-epidemiological data, performed from the blood of healthy donors, shows a prevalence of anti-*Parachlamydia* antibodies lower than 5%, and *P. acanthamoebae* was found to have a prevalence of ~4% in cases of community-acquired pneumonia in Switzerland ([5], unpublished data). Similarly, in 2017–2018 in southeastern Finland, it was detected by PCR in 5.8% of samples taken from patients ranging from 6 to 17 years old [43]. However, the prevalence in human lung infections may vary largely over time and location. This may be due to the many factors that impact exposure to this pathogen such as the prevalence in man-made water systems like humidifiers, climatization systems, decorative fountains, hot tubs and nebulizers similarly to other waterborne pathogens like *Legionella* and environmental mycobacteria [5,19, 20]. In addition to studies in Germany and Finland, the bacterium has been detected in France, Switzerland and Japan indicating a wide-ranging ecological niche [2,12, 34, 55].

### Prevention

There is no current prevention method that is recommended, but as it is likely transmitted by amoebae, avoiding areas with large amoebal populations may be the best prevention. Even so, it was also detected in hospital environments, so more data is needed to truly understand how and from where the pathogen can spread [55]. Prevention of exposure to *Parachlamydia* therefore relies on the quality of the man-made water system and chlorination, similar to measures aiming at reducing the burden of *Legionella* disease. Zoonotic risk remains to be determined.

## Open questions

1. Which virulence factors does *P. acanthamoebae* use to cause pneumonia, and does the infection work synergistically with other pathogens?
2. Is *P. acanthamoebae* infectious from person to person, or does it enter the person through the environment? If through the environment, which conditions are optimal for growth and transmission?
3. Does *P. acanthamoebae* cause infection via infected amoebae, or does it infect human cells as well? If through amoebae, what is the diversity of amoebae species that can host the bacterium?
4. How does *P. acanthamoebae* interact with the human immune system?
5. What is the incidence and prevalence of *P. acanthamoebae* in humans, and what is the impact of immunosuppression on the outcome of an infection?

## Abbreviations

BSL2: biosafety level two
CPAF: *Chlamydia* protease-like activity factor
EB: elementary body
Incs: inclusion membrane proteins
qPCR: quantitative PCR
QRDRs: quinolone resistance-determining regions
RB: reticulate body
T3SS: type III secretion system
